# Dissecting genetic links between Alzheimer’s disease and type 2 diabetes mellitus in a systems biology way

**DOI:** 10.3389/fgene.2022.1019860

**Published:** 2022-09-16

**Authors:** Peiyuan Kang, Zhao Wang, Dan Qiao, Bohan Zhang, Chenyu Mu, Huixian Cui, Sha Li

**Affiliations:** ^1^ Clinical Medicine, Hebei Medical University, Shijiazhuang, China; ^2^ Department of Anatomy, Hebei Medical University, Shijiazhuang, China; ^3^ Neuroscience Research Center, Hebei Medical University, Shijiazhuang, China; ^4^ Hebei Key Laboratory of Neurodegenerative Disease Mechanism, Shijiazhuang, China; ^5^ The Key Laboratory of Neural and Vascular Biology, Ministry of Education, Hebei Medical University, Shijiazhuang, China

**Keywords:** Alzheimer’s disease, type 2 diabetes mellitus, differentially analysis, semantic similarity, protein interaction

## Abstract

**Background:** Alzheimer’s disease (AD) and Type 2 Diabetes Mellitus (T2DM) are two of the most common diseases for older adults. Accumulating epidemiological studies suggest that T2DM is a risk factor for cognitive dysfunction in the elderly. In this study, we aimed to dissect the genetic links between the two diseases and identify potential genes contributing the most to the mechanistic link.

**Methods:** Two AD (GSE159699 and GSE28146) and two T2DM (GSE38642 and GSE164416) datasets were used to identify the differentially expressed genes (DEGs). The datasets for each disease were detected using two platforms, microarray and RNA-seq. Functional similarity was calculated and evaluated between AD and T2DM DEGs considering semantic similarity, protein-protein interaction, and biological pathways.

**Results:** We observed that the overlapped DEGs between the two diseases are not in a high proportion, but the functional similarity between them is significantly high when considering Gene Ontology (GO) semantic similarity and protein-protein interactions (PPIs), indicating that T2DM shares some common pathways with AD development. Moreover, we constructed a PPI network consisting of AD and T2DM DEGs, and found that the hub gene SLC2A2 (coding transmembrane carrier protein GLUT2), which connects the most DEGs in both AD and T2DM, plays as a key regulator in linking T2DM and AD *via* glucose metabolism related pathways.

**Conclusion:** Through functional evaluation at the systems biology level, we demonstrated that AD and T2DM are similar diseases sharing common pathways and pathogenic genes. SLC2A2 may serve as a potential marker for early warning and monitoring of AD for the T2DM patients.

## Introduction

Alzheimer’s disease (AD) is a chronic neurodegenerative disorder and the most common form of dementia that affects over 55 million people worldwide in 2020 (Gilbert, 2013). During the past decades, although massive efforts have been made to decipher the pathogenesis of AD, no effective therapies have been developed to tackle this complex neurodegenerative disease (Matthews et al., 2019). Two major pathological hallmarks were identified for AD, both involved neuronal apoptosis: β-amyloid plaques formed by toxic Aβ deposition and, neurofibrillary tangles (NFTs) caused by hyperphosphorylation of tau proteins (Marques et al., 2010).

Several risk factors that may trigger or facilitate the development of AD have been identified, including high cholesterol and Type 2 diabetes mellitus (T2DM) (Dominguez et al., 2012; De Felice et al., 2014). In recent years, accumulative evidence suggests shared pathology or treatment between T2DM and AD (Akter et al., 2011). T2DM, characterized by insulin resistance and relative insulin deficiency, is a disease of elderly persons with an increased risk of dementia at 1.5∼2.5 times (Exalto et al., 2012). Cohort studies also verified that T2DM is associated with late-onset AD. The most possible mechanism by which T2DM may contribute to the pathogenesis of AD is the alteration of insulin signaling in the brain. Insulin, a neuroprotective growth factor in the brain, could be desensitized in both diseases. Not only insulin could affect Aβ production and degradation, but also many downstream molecules in the insulin signaling pathway, such as GSK3β, ERK, AKT, etc., are involved in tau hyperphosphorylation (Takeda et al., 2011). Due to the close association between T2DM and AD, it is possible that drugs developed to treat T2DM, which targets insulin signaling, may be applied to prevent or suspend neuronal apoptosis in AD brain and lead to less cognitive impairment in AD patients.

Over the past decade, a growing number of transcriptome works have been conducted to identify expression alterations associated with complex diseases, which is a typical workflow in bioinformatics analysis and preclinical research (Santiago et al., 2019; Shu et al., 2022). But it is worth noting that many differentially expressed genes (DEGs) do not have known biological effects, or each gene may contribute small but complex effects to the pathogenesis of diseases (Riancho, 2012). According to a recent network-based study, genes that were differentially expressed in the disease condition tend to form modules of interacting and functionally related genes and propagate the effects of disease phenotype through a highly interconnected protein-protein interaction (PPI) network. In other words, DEGs may indirectly work together with others involved in the same pathways or implemented in similar biological processes.

Among elderly people, co-morbidity is an increasingly common medical reality (Tabares-Seisdedos and Rubenstein, 2013). Despite unique pathological features of each disease, some essential cellular functions or molecular processes whose alterations might collectively dictate disease progression are similar to the other types of diseases. In this study, we systematically compared the molecular mechanisms and relationships of AD and T2DM by integrating transcriptome data, interactome data, and function data, to examine the existence of shared risk for AD and T2DM. The consistency of these two diseases was evaluated at the functional similarity and gene interaction levels. Although many studies have indicated the underlying links between AD and T2DM, our study comprehensively investigates their connections from the perspective of functional analysis and interaction analysis. Also, SLC2A2 was detected as the crosstalk gene playing a key role in linking these two diseases for further experimental validation.

## Materials and methods

### Gene expression data

The human gene expression data of AD and T2DM were collected from the Gene Expression Omnibus (GEO) database (Barrett et al., 2013). For AD, GSE159699 includes 12 disease and 10 normal samples while GSE28146 data include 22 disease and 8 normal samples (Blalock et al., 2011; Nativio et al., 2020). For T2DM, GSE38642 consists of nine diseases and 54 normal islets samples while the GSE164416 contains 39 disease and 18 control islets samples, which are selected from 133 samples among four diabetes statuses (Taneera et al., 2012; Wigger et al., 2021). For each disease, RNA-seq and microarray platforms were separately used for the two datasets. Specifically, GSE159699 and GSE164416 were detected using RNA-seq platform while GSE28146 and GSE38642 were measured using microarray platform. The platform information is detailed in Table 1.

**TABLE 1:** AD and T2DM gene expression datasets.

Dataset	Disease	Tissue	Technology	Platform	Sample number		
					Total	Case	Normal
GSE159699	AD	Hippocampus	RNA-seq	Illumina NextSeq 500	30	12	10
GSE28146	AD	Hippocampus	Microarray	Affymetrix Human Genome U133	30	22	8
				Plus 2.0 Array			
GSE38642	T2DM	Pancreas	Microarray	Affymetrix Human Gene 1.0 ST Array	63	9	54
GSE164416	T2DM	Pancreas	RNA-seq	Illumina HiSeq 2500	57	39	18

### Data preprocessing and differential analysis

The Affymetrix GeneChip data are preprocessed by RMA (Robust Multi-array Analysis) (Bolstad et al., 2003). The annotation soft tables downloaded from the corresponding GPL platform were used for assigning Probe ID to Gene Symbol. Probes with ambiguous or multiple gene symbols were removed. Averaged the expression intensity when replicated probes mapping to the identical gene. The entire expression matrix was log2 transformed. Fold Change (FC) and Mann-Whitney test were used to identify the differentially expressed genes (DEGs). Genes with |FC|>1.5 and Mann-Whitney test *p*-value<0.05 were defined as DEGs. Considering that different array platforms have different gene coverage, we just studied genes presented in all the analyzed datasets. Reads per kilo base per million mapped reads (FPKM) was used to measure the expression intensity for the RNA-seq data and the data was preprocessed using the methods mentioned in the original papers. All the analysis was carried out using R-4.1.2.

### Protein interaction data

The human protein-protein interaction (PPI) data were derived from the Search Tool for the Retrieval of Interacting Genes/Proteins (STRING version 11.5) (Szklarczyk et al., 2019). Interaction with a score greater than 0.4 were used to build a high-confidence network with 38 edges and 166 nodes. Network degree was defined as the number of neighbors linking a protein. PPI network was generated and illustrated by Cytoscape (version 3.9.1) (Shannon et al., 2003).

### Monte carlo simulation

Monte Carlo simulation, also known as multiple probability simulation, is a mathematical technique used to estimate the possible outcomes of an uncertain event. *p*-values are calculated to see whether observed values are unusually large or small for the null distribution. This calculation compares the observed value to the upper/lower tails of the null distribution to explore whether the observed value is significantly large/small for the distribution.

p=(m+1)/(n+1) (1)

where n is the total number of Monte Carlo simulations, m is the number of simulations for which the statistic was greater than or equal to the observed statistic. One (1) is added to the numerator and denominator because the observed statistic is included in the reference distribution.

In this study, to access the statistical significance of an observed SS score, we randomly selected two gene sets with the same sizes as the two original sets from the background genes detected by both diseases. We then calculated *p*-value using these two gene sets. After 10,000 Monte Carlo random experiments, the significance level (or *p*-value) for an observed SS was calculated as the proportion of random scores higher than the observed score.

### Pathway enrichment analysis

The hypergeometric distribution model was used to evaluate the significance of enrichment analysis. The probability of observing at least k genes annotated in a specific term is calculated as follows:

$$p=1-\overset{q-1}{\underset{k=0}{\sum }}\frac{\left(\begin{array}{c} t\\ k \end{array}\right)\left(\begin{array}{c} m-t\\ n-k \end{array}\right)}{\left(\begin{array}{c} m\\ n \end{array}\right)}$$ (2)

where k is the number of genes of interest, n is the total number of detected genes, m is the term size or the number of genes in a term, and t is the number of the overlap genes annotated in the term. The resulting *p*-value was then multi-test adjusted by the BH correction (FDR< 0.05).

Functional enrichment analysis of Gene Ontology (GO) (Gene, 2021) and Kyoto encyclopedia of genes and genomes (KEGG) (Kanehisa et al., 2021) pathway was performed to determine significantly enriched gene functions using the R package ‘clusterpProfiler’ (R version 4.1.2) (Yu et al., 2012). Three pathways, Glucagon Signaling Pathway, Carbohydrate Digestion and Absorption and Central Carbon Metabolism in Cancer, are collected and highlighted from KEGG.

Five semantic similarity methods were used to evaluate the functional similarity between two gene sets, i.e., Wang, Rel, Jiang, Lin and Resnik (Yu et al., 2012). ‘GOSemSim’ was used for the calculation of these scores.

## Results

### Differential analysis

**FIGURE 1:**
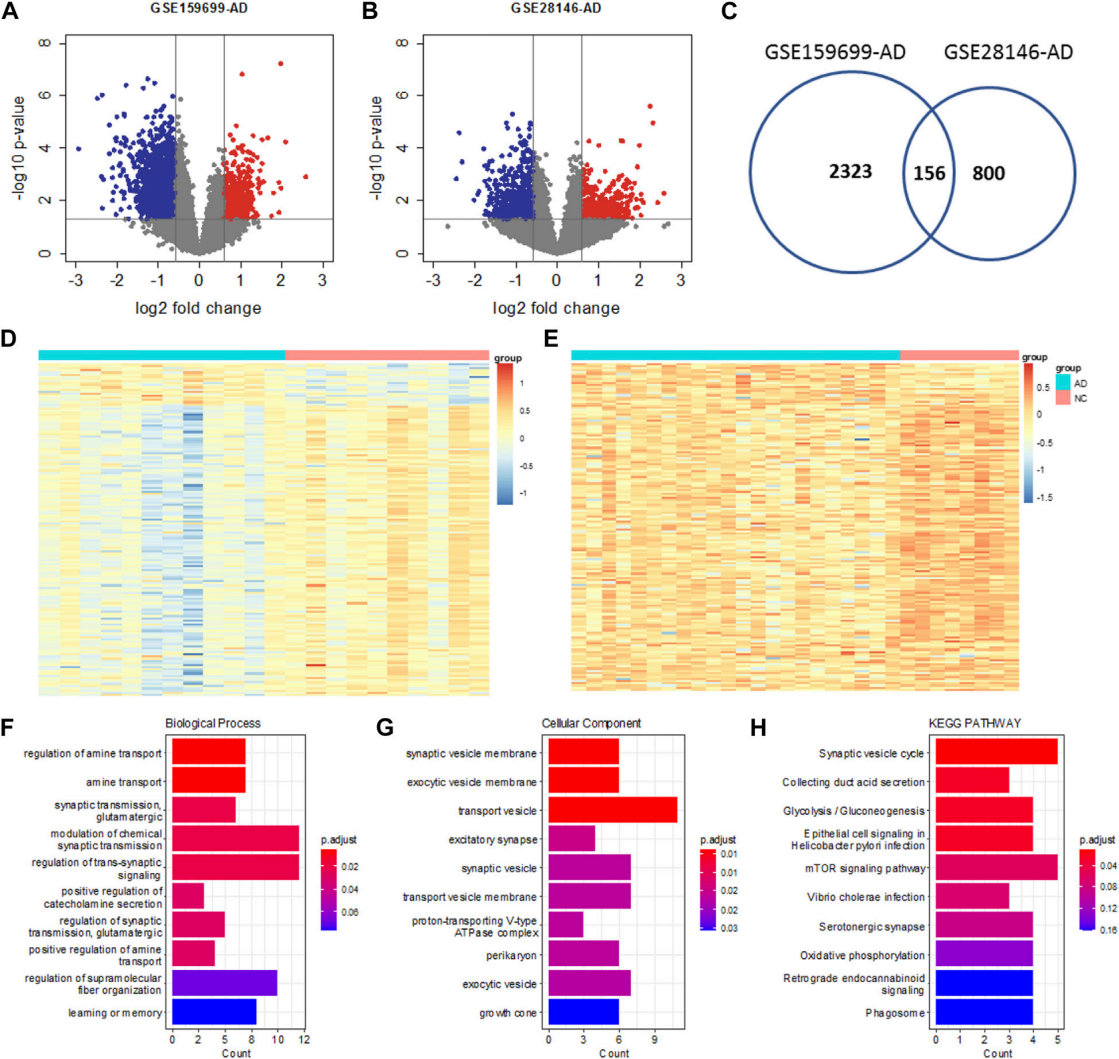
Differential analysis of AD. Volcano plot of GSE159699 **(A)** and GSE28146 **(B)**. DEGs are defined as genes with |FC|>log_2_(1.5) and *p*-value<0.05. **(C)** Venn diagram showing the intersection between DEGs of GSE159699 and GSE28146. Heatmap of expression changes for the common AD DEGs in GSE159699 **(D)** and GSE28146 **(E)**. **(F**–**H)** Functional enrichment analysis of the common AD DEGs using Biological Process, Cellular Component and KEGG Pathway.

Datasets GSE159699 and GSE28146 were used to identify the differentially expressed genes (DEGs) for AD. Genes with fold change (FC) larger than 1.5 and *p*-value less than 0.05 were identified as DEGs for subsequent analysis. In total, 2323 and 800 DEGs were identified for GSE159699 and GSE28146 (Figures 1A,B), respectively, in which 156 were commonly detected as DEGs (Figure 1C) and hereafter we defined it as AD DEGs. For these genes, the expression difference between the AD and control samples are shown in Figure 1D and Figure 1E for GSE159699 and GSE28146, respectively, which illustrates that the two groups of samples can be clearly stratified.

These genes are mainly involved in biological processes of regulation of amine transport, regulation of trans-synapic signaling, learning of memory, etc. (Figure 1F) and locate in transport vesicle, synaptic vesicle, synaptic vesicle membrane, etc. (Figure 1G). Also, they are implemented in biological pathways of synaptic vesical cycle, collecting duct acid secretion, glucolysis, etc. (Figure 1H).

**FIGURE 2:**
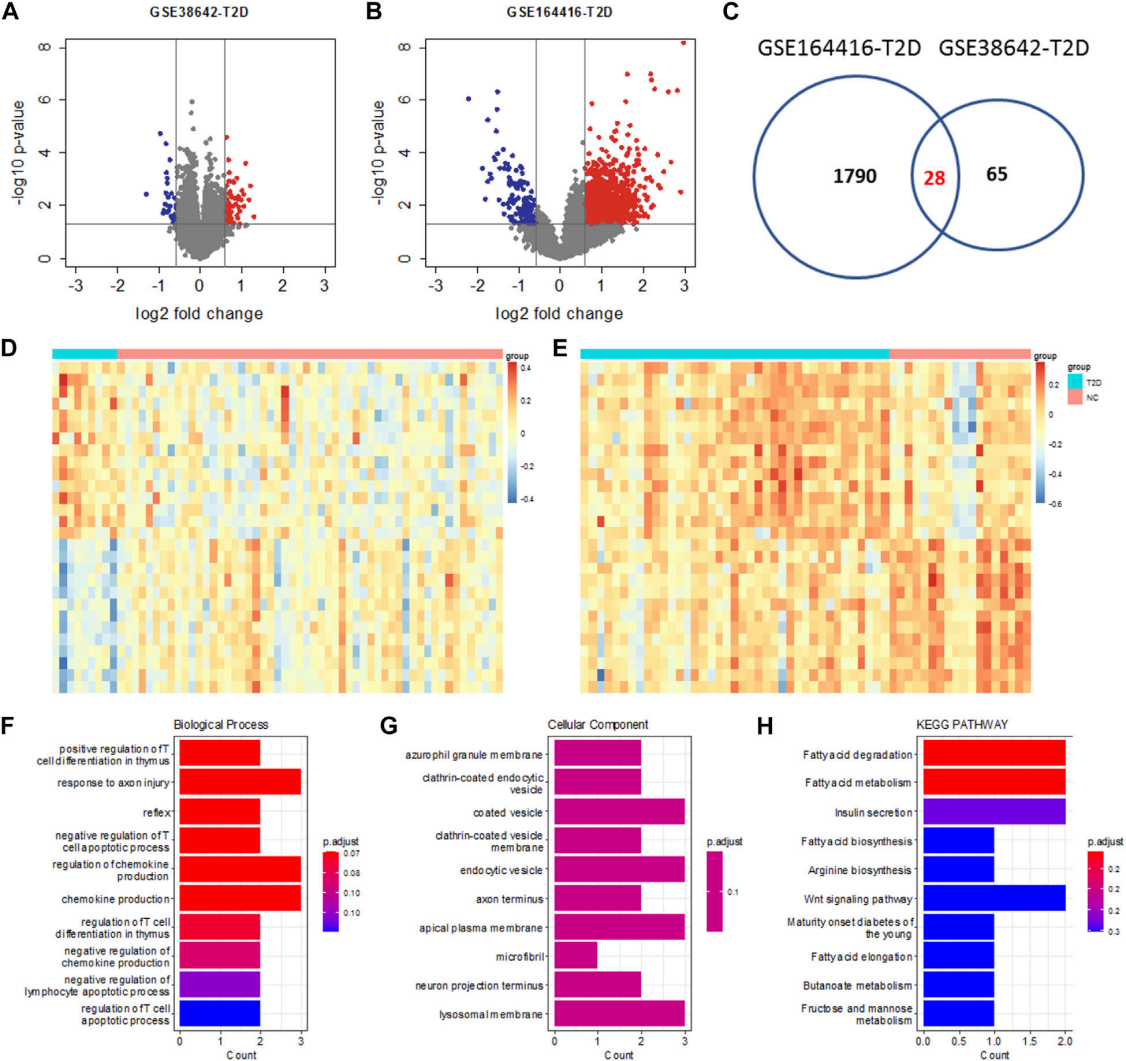
Differential analysis of T2DM. Volcano plot of GSE38642 **(A)** and GSE164416 **(B)**. The thresholds for the identification of T2DM DEGs are the same as AD. **(C)** Venn diagram showing the intersection between DEGs of GSE38642 and GSE164416. Heatmap of expression changes for the common T2DM DEGs in GSE38642 **(D)** and GSE164416 **(E)**. **(F**–**H)** Functional enrichment analysis of the common T2DM DEGs using Biological Process, Cellular Component and KEGG Pathway.

Datasets GSE164416 and GSE38642 were used to identify DEGs for T2DM. 1790 and 65 DEGs were identified for GSE159699 and GSE28146 (Figures 2A,B), respectively. The 28 DEGs commonly identified from them were T2DM DEGs (Figure 2C). For these genes, the expression difference between the T2DM and control samples is clear based on these T2DM DEGS for GSE164416 and GSE38642 (Figures 2D,E).

These genes are enriched in biological processes of positive regulation of T cell differentiation in thymus, regulation of chemokine production regulation of lymphocyte apoptotic process, etc. (Figure 1F) and resident in azurophil granule membrane, coated vesicle, endocytic vesicle, lysosomal membrane, etc. (Figure 1G). Also, they are implemented in biological pathways of fatty acid metabolism, insulin secretion, arginine biosynthesis, etc. (Figure 1H).

### Functional analysis

**FIGURE 3:**
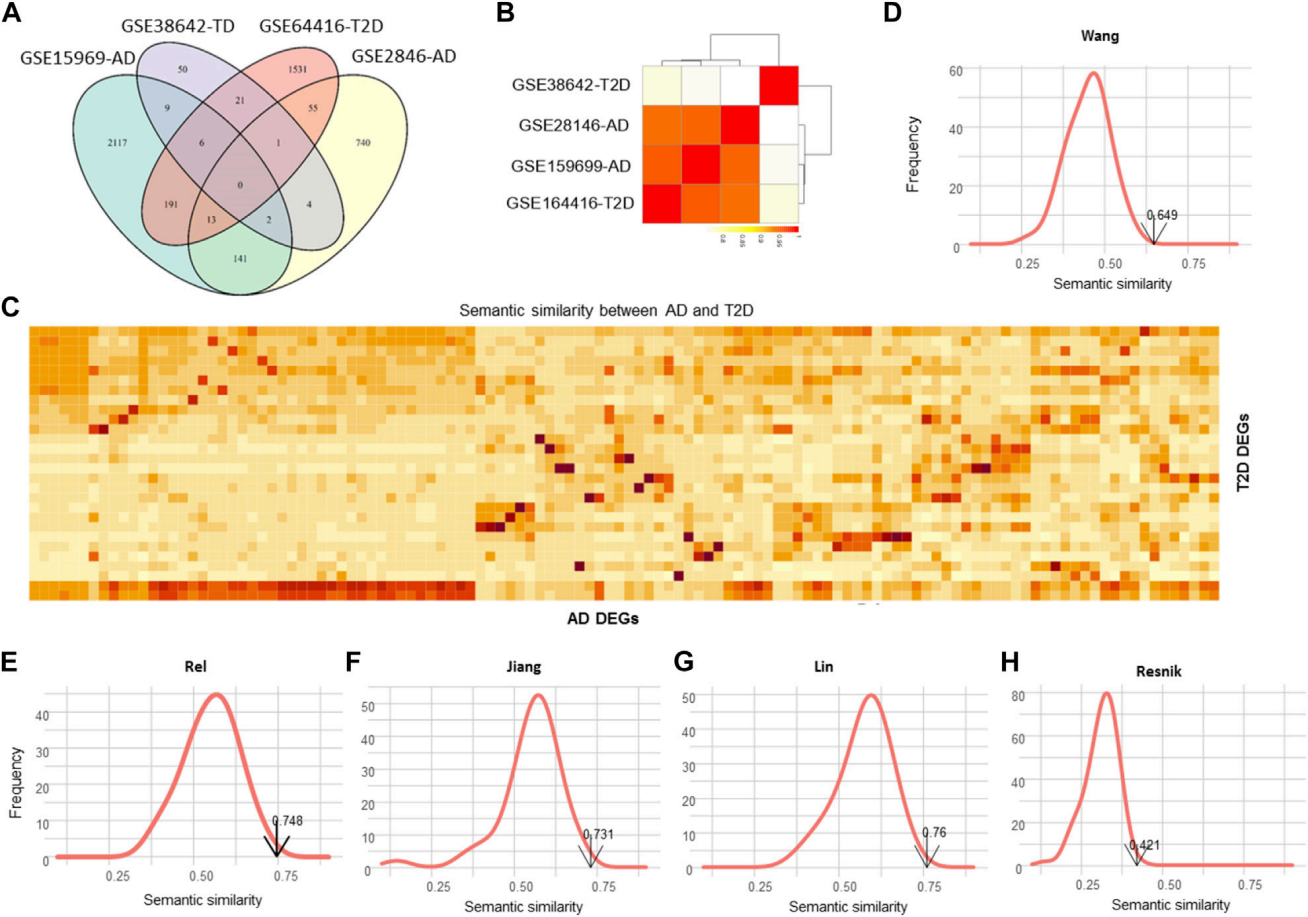
Evaluation of the functional similarity between AD and T2DM. **(A)** Venn diagram illustrating the DEGs identified from GSE159699, GSE28146, GSE38642 and GSE164416. **(B)** Semantic similarity among the DEGs of the four datasets. **(C)** Heatmap showing the semantic similarity between AD and T2DM DEGs. **(D**–**H)** Distributions of the simulated semantic similarity for Wang, Rel, Jiang, Lin and Resnik. The simulated scores are generally less than the detected one.

Dozens of DEGs were commonly detected by different datasets for each disease, whereas no common DEGs were identified between AD and T2DM (Figure 3A), given that the two diseases are strongly associated. However, these DEGs are closely related to each other from the function perspective. Specifically, the semantic similarity (SS) scores among the four datasets are generally over 0.8, especially for the three datasets GSE28146, GSE159699 and GSE164416 (SS > 0.9, Figure 3B). Figure C shows the SS scores between the 156 AD DEGs and 28 T2DM DEGs. Using Monte Carlo simulation, we randomly selected the same number of DEGs 1,000 times and calculate the SS scores to build a simulated SS distribution. The detected SS score between the 156 AD DEGs and 28 T2DM DEGs is 0.649, which is significantly higher than most of the simulated ones (*p* < 0.01, Figure 3D), indicating that AD and T2DM are close to each other from the point of biological function.

Using the other four SS methods, also, we calculated the SS scores and simulated SS distributions (Figures 3E–H), resulting in the SS scores of 0.748, 0.732, 0.76 and 0.421 for Rel, Jiang, Lin and Resnik, respectively. As expected, the SS scores of the simulated data are consistently lower than the real SS scores (*p* < 0.01), demonstrating that the DEGs of the two diseases are involved in some common pathways or functional modules.

### Network analysis

**FIGURE 4:**
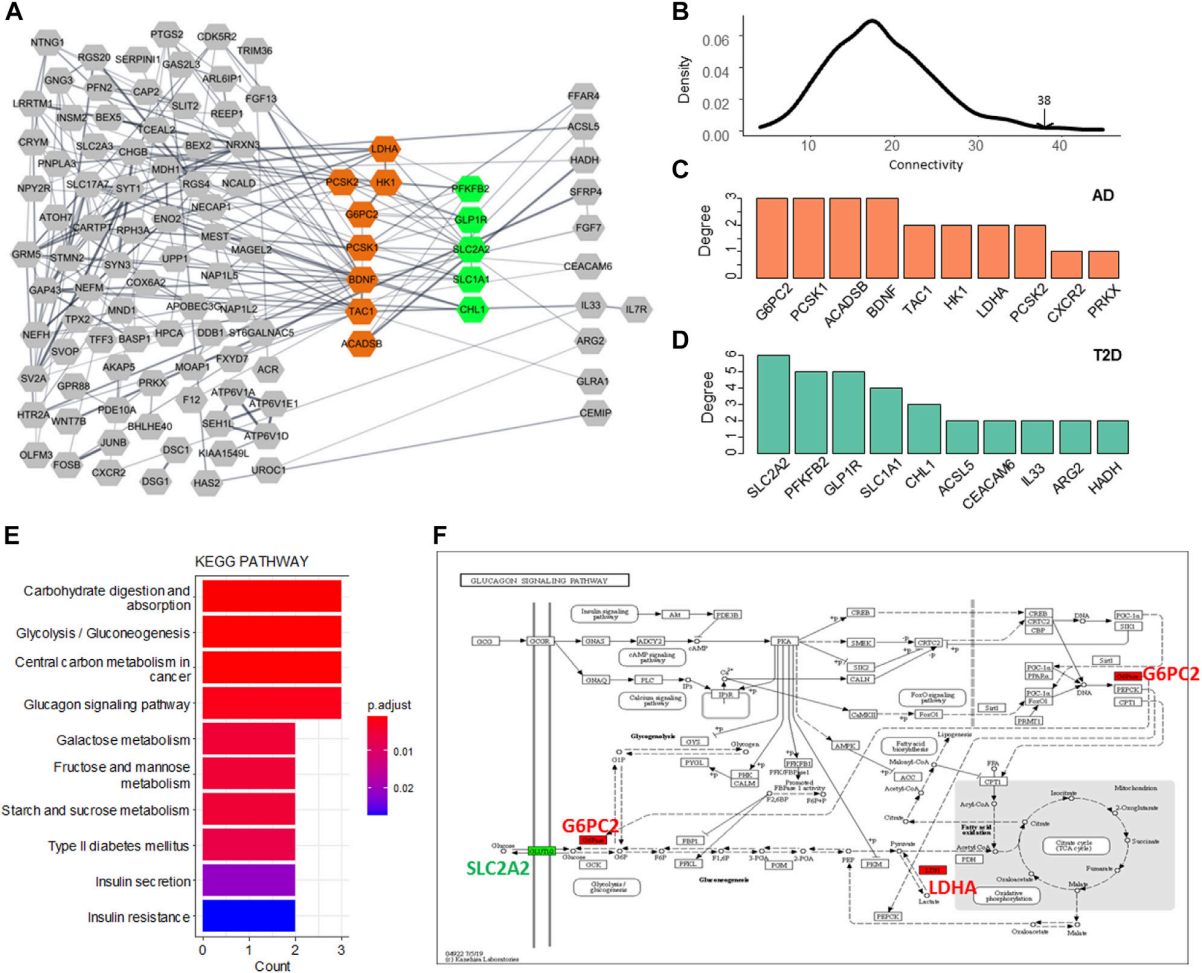
Network analysis of the AD and T2DM DEGs. **(A)** PPI network of the AD and T2DM DEGs. Node represents DEG while edge indicates interaction between a pair of DEGs. The hub genes of AD and T2DM are highlighted in orange and green, respectively. **(B)** Distribution of the connectivity between simulated data. **(C**,**D)** Hub genes with top network degree for AD and T2DM, respectively. **(E)** Enriched KEGG pathways of the 13 hub DEGs. **(F)** Glucagon signaling pathway. G6PC2 and LDHA (AD DEGs) are colored in red and SLC2A2 (T2DM DEG) is colored in green.

To investigate the associations between the two diseases from the point of protein interaction, we constructed a protein-protein interaction (PPI) network using the AD DEGs and T2DM DEGs. A network consisting of 184 genes, 156 AD DEGs and 28 T2DM DEGs, was illustrated in Figure 4A and the genes with high degree were highlighted (AD in orange and T2DM in green). 38 edges were observed connecting the two gene set. To test whether the two gene sets are closely connected with each other, we randomly selected the same number of genes for each set 1,000 times and calculated the connectivity between them. Distribution of the random data was built and only a few simulations were larger than 38 (Figure 3B).

**FIGURE 5:**
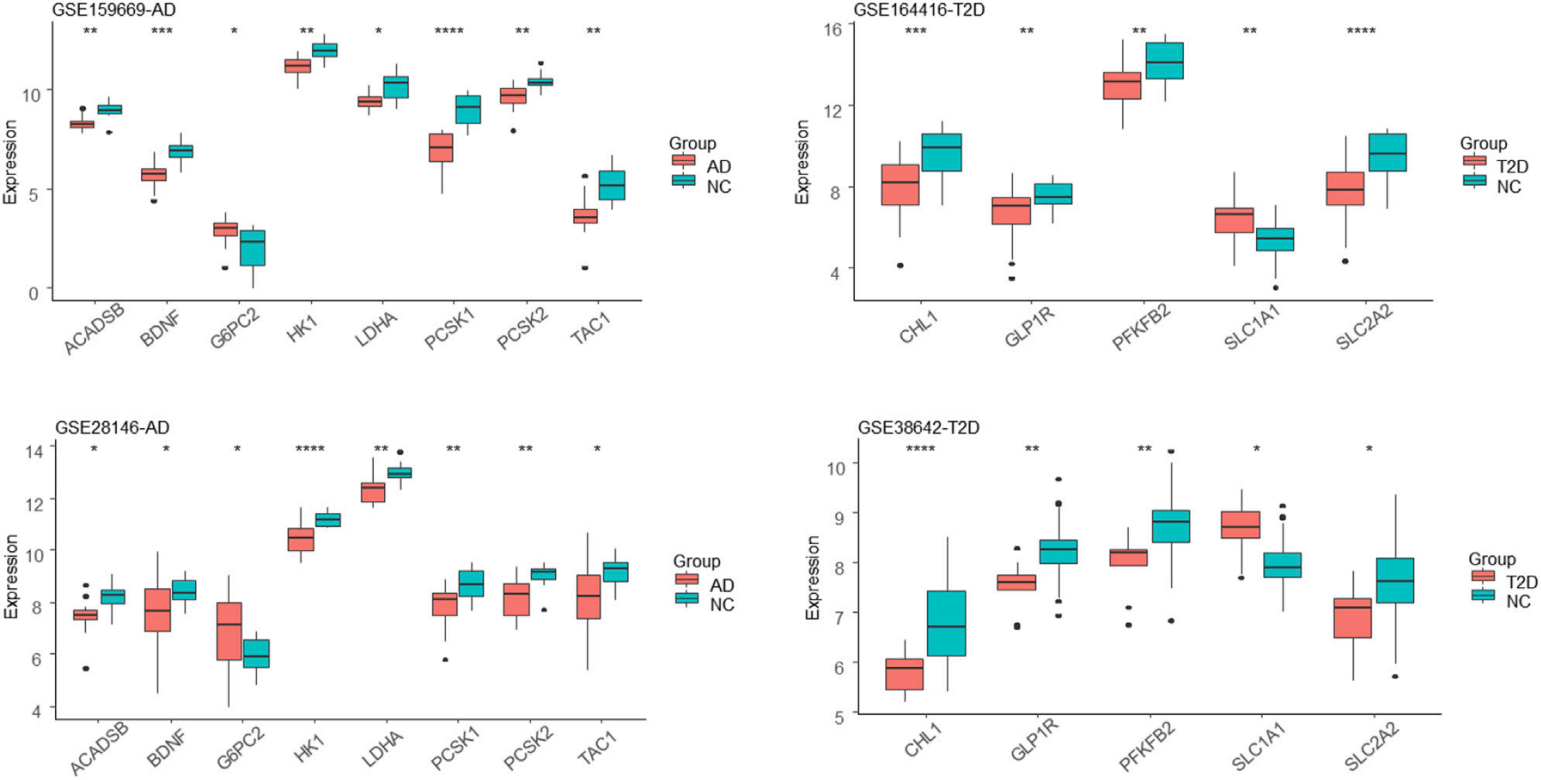
Expression distribution of the hub DEGs. AD and T2DM groups are colored in pink and the corresponding control groups are colored in cyan. *, **, ***, and **** represent the Mann-Whitney test *p*-value less than 0.05, 0.01, 0.001 and 0.0001, respectively.

The hub genes or genes with the highest degree for AD and T2DM were shown in Figures 4C,D, respectively. The AD hub genes consist of G6PC2, PCSK1, ACADSB, and BDNF, while SLC2A2, PFKFB2, GLP1R, SLC1A1, and CHL1 are the hub genes of T2DM. It is apparent that the hub DEGs are connected much denser than the other DEGs, suggesting they are involved in biological pathways linking AD and T2DM, such as carbohydrate digestion and absorption, glycolysis, glucagon signaling pathway, etc. (Figure 4E). Among these hub genes, SLC2A2, a T2DM hub gene, was up-regulated and all the others were down-regulated. Also, it has the largest connectivity of 6 and connects most of the AD hub genes, suggesting that it is a pivot mediating or triggering the development from T2DM to AD. Additionally, we observed that most of the hub DEGs were down-regulated, seven out of eight in AD and four out of five in T2DM (Figure 5).

### Pathway analysis

The glucagon signaling pathway is a process of a series of elevated blood glucose enzymatic reactions triggered by the binding of glucagon that produced by pancreatic islets alpha cells to the glucagon receptor on the surface of liver cells. Glucagon signaling pathway mainly assists glucagon to exert its role of raising blood glucose to sustain blood glucose homeostasis in the body and synergizes with insulin. In this pathway, GLUT2, encoded by SLC2A2, is working as a transmembrane carrier protein that enables protein facilitate glucose movement across cell membranes. Two AD hub genes/proteins, G6PC2 and LDHA that are interacted with GLUT2, are implemented in the pathway (Figure 4F).

**FIGURE 6:**
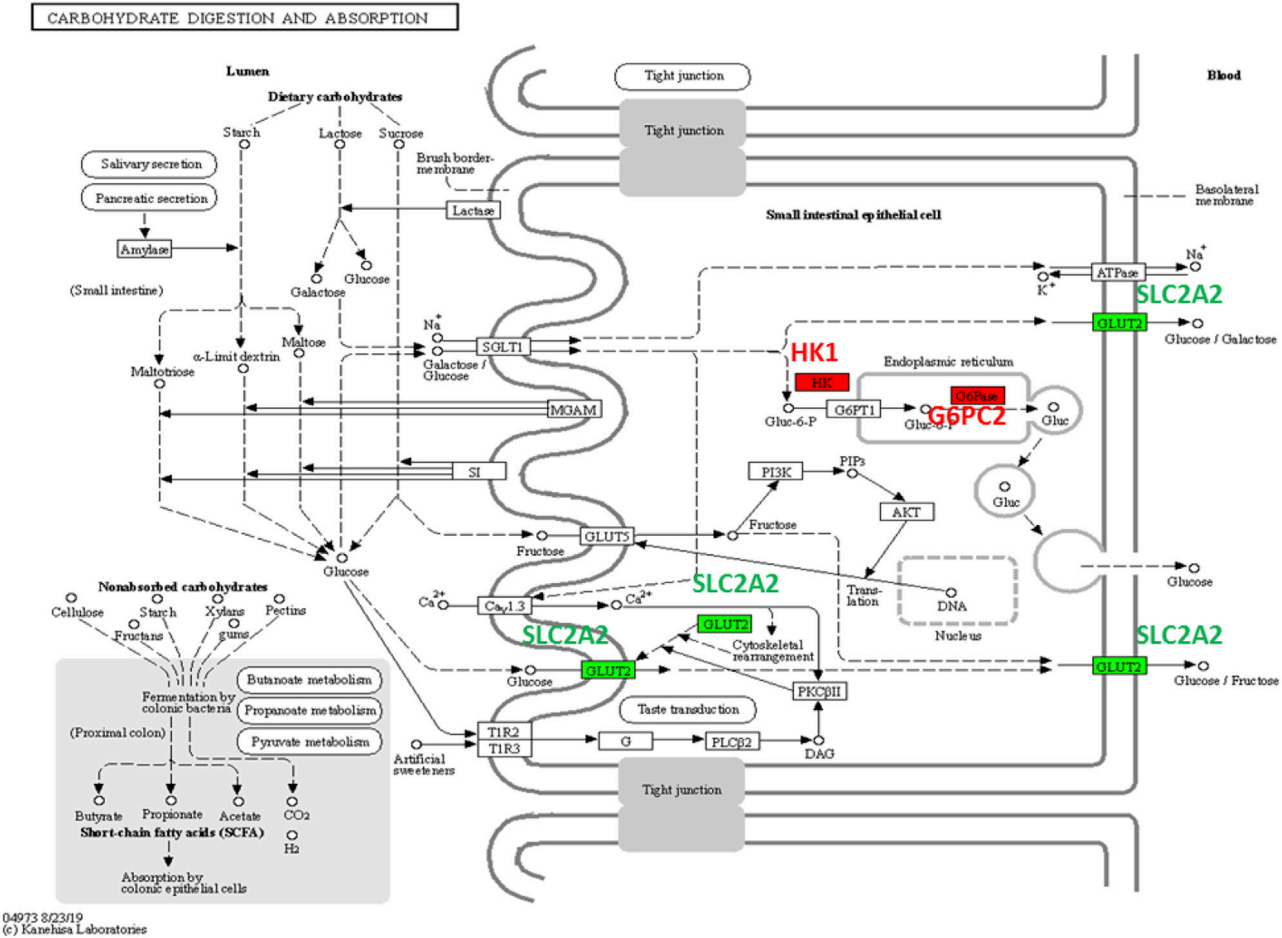
Carbohydrate digestion and absorption pathway. G6PC2 and HK1 (AD DEGs) are colored in red and SLC2A2 (T2DM DEG) is colored in green.

In the carbohydrate digestion and absorption pathway, GLUT2 is critical and ubiquitous in carbohydrate transport (Figure 6). Glucose and galactose are initially transported into the enterocyte by SGLT1 located in the apical brush border membrane (BBM) and then exit through the basolateral membrane by GLUT2 or release out through exocytosis by HK1 and G6PC2 located in Endoplasmic Reticulum (ER). In intestinal glucose absorption, transport by SGLT1 induces rapid insertion and activation of GLUT2 in the BBM by a PKCβII-dependent mechanism. Moreover, trafficking of apical GLUT2 is rapidly promoted by glucose, which acts through T1R2 + T1R3/alpha-gustducin to activate PLCβ2 and PKCβII.

## Discussion

We dissected the genetic links between Alzheimer’s Disease (AD) and Type 2 Diabetes Mellitus (T2DM) in a systems biology way. The differentially expressed genes between the two diseases are not highly overlapped, but the functional similarity between them is significantly high when considering Gene Ontology semantic similarity and protein-protein interactions, indicating that AD and T2DM share some common pathways in disease development. From the interaction network of DEGs (Figure 4A), SLC2A2, coding transmembrane carrier protein GLUT2, has the highest connectivity with other DEGs for both AD and T2DM. According to these observations, we suspected SLC2A2 is a potential contributor linking T2DM and AD via glucose metabolism related pathways.

Glucose uptake mediated by GLUTs is the first step of glucose metabolism (van der Velpen et al., 2019). Glucose metabolism entails both delivery of glucose to cells from the bloodstream, and converting into adenosine triphosphate (ATP) taking place in mitochondria. Early changes to glucose metabolism possibly result from abnormal delivery of glucose to the brain. Glucose is virtually the sole fuel for your brain, which is a hydrophilic molecule and requires protein transporters to cross cell membranes. Glucose is released into the bloodstream and taken up by the brain via the sodium-independent facilitative transporters GLUT1 and GLUT3.

GLUT1 is responsible for glucose uptake across the BBB endothelial cells and into astrocytes. Glucose uptake into the brain appears to correlate with the number of GLUT1 transporters at the BBB. Reports showed that neurons do not express GLUT1 (Zlokovic, 2011), and GLUT3 is the key glucose transporter that promotes the uptake of glucose into neurons (Patching, 2017). Iadecola et al. demonstrated that a decrease of glucose in the brain *via* loss of these major glucose transporters may reduce brain glucose and therefore limit the metabolism processes (Iadecola, 2015). According to recent brain studies, most glucose transport is regulated by GLUT1 and GLUT3, but Knezovic et al. evidenced that GLUT2 leads specific neuronal populations more vulnerable to pathogenic mechanisms underlying AD (Knezovic et al., 2017).

The influence of the demographic characteristics among the four datasets was not evaluated in this study, due to only the two microarray datasets have demographic characteristics while the two RNA-seq datasets do not have. For the T2DM dataset GSE38642, 36 male and 27 female samples are included with an average age of 57. For the T2DM dataset GSE28146, 12 male and 18 female samples are included with an average age of 85. The AD patients are generally much older than the T2DM patients, although both T2DM and AD are characterized by increased incidence and prevalence with aging.

Through functional evaluation at the systems biology level, we demonstrated that AD and T2DM are similar diseases sharing common pathways and pathogenic genes. SLC2A2 may serve as a potential marker for early warning and monitoring of AD for the T2DM patients.

## Data Availability

The datasets presented in this study can be found in online repositories. The names of the repository/repositories and accession number(s) can be found in the article/supplementary material.
